# The Work of the Holy Ghost

**Published:** 1887-06-18

**Authors:** 


### Words of Consolation.

[Communications for this column should be addressed, "Chaplain," care of the Editor of The Hospital, who will gratefully welcome any suggestions or inquiries from matrons, nurses, and the staff generally.]

### XXXVII.—The Work of the Holy Ghost.

"The Faith, once delivered to the Saints," has been handed down to us. However false or mistaken fallible teachers may be in their interpretations, the whole gospel truth is contained for us in the New Testament and the Creeds of the Church. The Holy Spirit, working upon the minds of the Apostles and Evangelists, helped them to formulate, then to teach and write what God would have us believe and do. Thus our Lord's words have been fulfilled. "He shall not speak of himself, but whatsoever he shall hear, that shall he speak; and he will show you things to come. He shall glorify me, for he shall receive of mine, and shall show it unto you" (John xvi, 13, 14). The guidance of the Spirit has been further manifested in the gradual recognition by the Church of the Canonical books of Holy Scripture. The Bible did not drop down upon us from heaven. The Old Testament was, of course, completed, and most jealously guarded by the Jewish Church, but the books which form the New Testament were many of them letters written, either to individuals, or to different congregations, and only came to be generally received as inspired writings by the whole Church as time went on. Other gospels and other epistles were written besides those which now compose the New Testament. Some of these are still extant. The New Testament became, therefore, a living witness to the work of the Holy Spirit in the Church, long after the first teachers of the gospel were dead. The sifting of the inspired from the uninspired writings of the Early Church was a gradual process. "It is possible (says Professor Westcott) that we might have wished much of this, or all this otherwise; we might have thought a Bible, of which every part should bear an unquestioned authentication of its divine origin, separated by a solemn act from the first, from the sum and fate of all other literature, would have best answered our conceptions of what the written records of revelation should be. But it is not thus that God works among us. In the Church, and in the Bible, alike He works through men. As we follow the progress of their formation, each step seems to be truly human, and when we contemplate the whole, we joyfully recognise that every part is also Divine."

We must also gratefully recognise the guidance and inspiration of the Holy Ghost in the liturgy of the Church. Our Prayer Book is not perfect, but no one can be familiar with those parts of it which are derived from the most ancient sources without feeling how intensely spiritual were the minds of the holy men who composed them. The necessary gifts for composing prayers seem now somewhat in abeyance. Seldom do modern compositions, when called for, approach either the beauty or the devotion, or the rhythm of the ancient models. Possibly the Holy Spirit is less active in this direction, in order that His guidance in the past may seem the more conspicuous and precious in our present heritage of the liturgy.

(*To be continued.*)

